# Most frequently asked questions about the coercivity of Nd-Fe-B permanent magnets

**DOI:** 10.1080/14686996.2021.1916377

**Published:** 2021-06-04

**Authors:** Jiangnan Li, Hossein Sepehri-Amin, Taisuke Sasaki, Tadakatsu Ohkubo, Kazuhiro Hono

**Affiliations:** Elements Strategy Initiative Center for Magnetic Materials (ESICMM), National Institute for Materials Science, Tsukuba, Japan

**Keywords:** Nd-Fe-B magnets, coercivity, thermal stability, microstructure, micromagnetic simulations, 50 Energy Materials, Permanent magnets, 203 Magnetics / Spintronics / Superconductors

## Abstract

Physically, the coercivity of permanent magnets should scale with the anisotropy field of ferromagnetic compounds, *H*_A_; however, the typical coercivity values of commercial polycrystalline sintered magnets are only ~0.2 *H*_A_, which is known as Brown’s paradox. Recent advances in multi-scale microstructure characterizations using focused ion beam scanning electron microscope (FIB/SEM), aberration corrected scanning transmission electron microscopy (C_s_-corrected STEM), and atom probe tomography (APT) revealed detailed microstructural features of commercial and experimental Nd-Fe-B magnets. These investigations suggest the magnetism of a thin layer formed along grain boundaries (intergranular phase) is a critical factor that influences the coercivity of polycrystalline magnets. To determine the magnetism of the thin intergranular phase, soft X-ray magnetic circular dichroism and electron holography play critical roles. Large-scale micromagnetic simulations using the models that are close to real microstructure incorporating the recent microstructure characterization results gave insights on how the coercivity and its thermal stability is influenced by the microstructures. Based on these new findings, coercivity of Nd-Fe-B magnets is being improved to its limit. This review replies to the most frequently asked questions about the coercivity of Nd-Fe-B permanent magnets based on our recent studies.

## Graphical abstract

**Figure:**
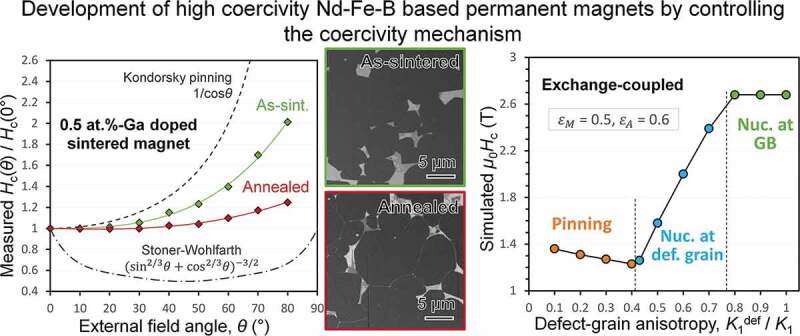

## 1. Introduction

Nd-Fe-B-based permanent magnets, independently invented by Sagawa [1] and Croat [2] in 1982, are one of the most important industrial materials that are used as magnetic field sources for motor, generator and actuators. It is foreseen that the needs for the high-performance Nd-Fe-B-based permanent magnets will increase further due to the rapid expansion of applications in green energy sectors such as motors and generators for electric vehicles, drones, robots, and wind turbines [3]. Although the maximum energy product of Nd-Fe-B is approaching its theoretical limit, the coercivity that is the most important extrinsic properties for permanent magnet application is still far below their theoretical limits. Physically, the coercivity of permanent magnets should scale with the anisotropy field of ferromagnetic compounds, *H*_A_; however, the typical coercivity values of commercial polycrystalline sintered magnets are only around 0.2 *H*_A_, which is known as Brown’s paradox. For example, the typical value of coercivity, µ_0_*H*_c_, for N50 type magnet with (*BH*)_max_~4 00 kJ/m^3^ is ~1.2 T, which is only ~15% of the anisotropy field of the Nd_2_Fe_14_B compound (µ_0_*H*_A_ ~ 7.5 T) [4,5]. Due to the thermal demagnetization effect, coercivity decreases to ~0.2 T at ~160°C, which is the operating temperature of the magnet in the traction motor of (hybrid) electric vehicles. The industrial solution for this was to substitute part of Nd in the Nd_2_Fe_14_B lattice with Dy to increase the anisotropy filed [4]. The resultant coercivity of (Nd_0.7_Dy_0.3_)-Fe-B magnets reaches 3.0 T. However, limited natural resources for Dy and its supply risks have raised research interest to develop high coercivity Dy-free Nd-Fe-B magnets with a better thermal stability of coercivity [6,7].

Coercivity is an extrinsic magnetic property and is strongly controlled by microstructure. Intensive researches have been carried out to pursue better understanding of the microstructure–coercivity relationships of Nd-Fe-B magnets. However, due to the limitations of the structural characterization techniques in 1980s, several questions remained unanswered until recently. In this paper, we review recent microstructure investigations and micromagnetic simulations and address answers to the most frequently asked questions about the coercivity of the Nd-Fe-B-based magnets. We will also address how to develop an optimum microstructure that leads to high coercivity without or with minimal use of heavy rare earth (HRE) elements.

## 2. Why is *H*_c_ of sintered magnets only 0.2*H*_A_?

**Figure 1.**
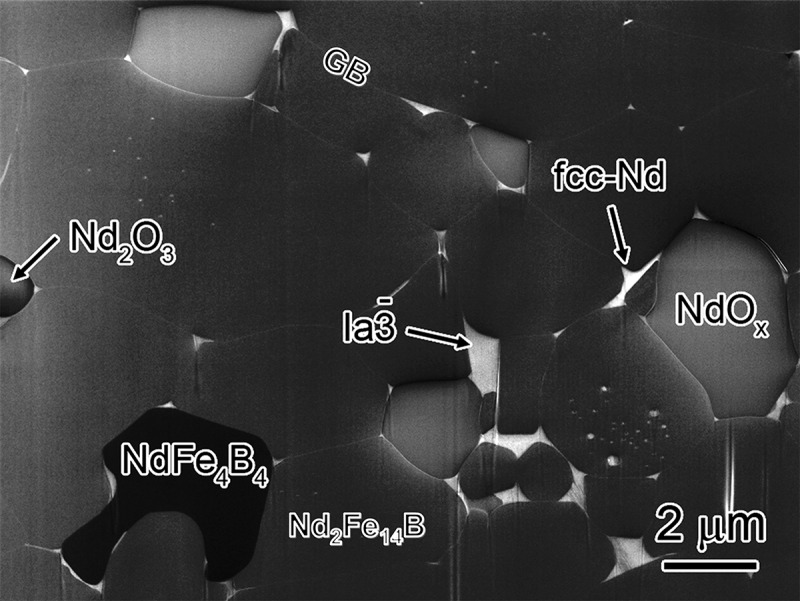
In-lens secondary electron SEM image showing general microstructure of typical Nd-Fe-B sintered magnet

**Figure 2.**
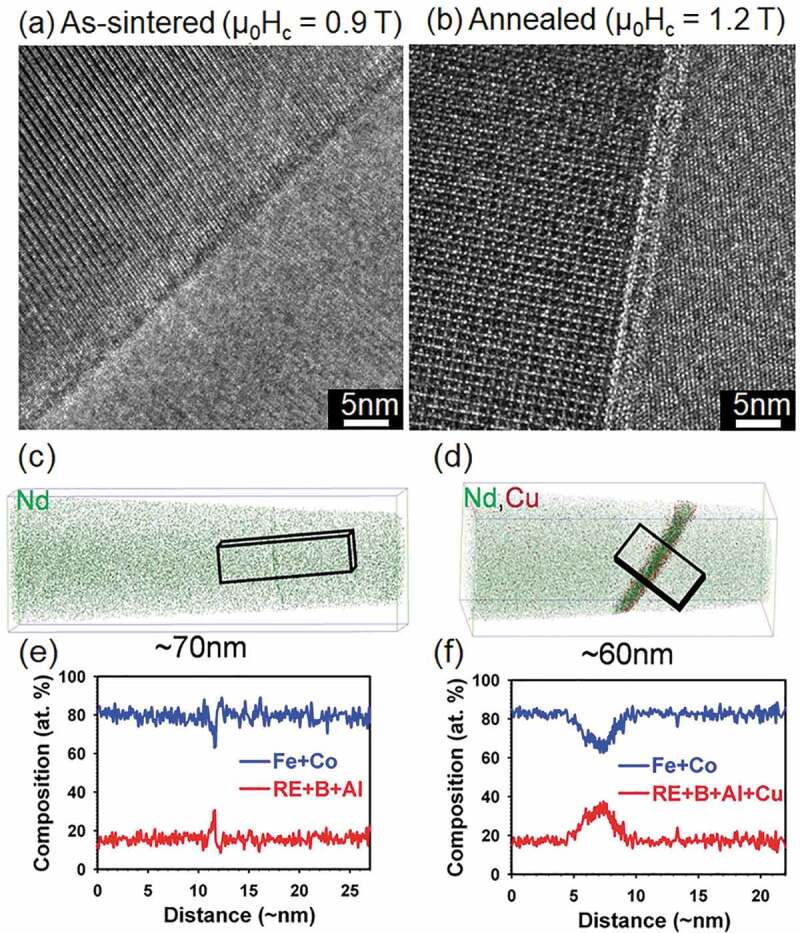
High-resolution TEM, atom probe tomography and composition line profile obtained from intergranular phase of (a, b) as-sintered and (c, d) optimally annealed Nd-Fe-B sintered magnet

Reproduced from Li et al. [19] and Sepehri-Amin et al. [21] with copyright permissions from Elsevier.

Nd-Fe-B sintered magnets are produced by a liquid sintering process. Magnetically aligned single crystalline fine particles are isostatically pressured to make a green compact followed by liquid sintering at an elevated temperature of ~950–1100°C. The Nd-Fe-B magnets right after the sintering process show a very small coercivity of ~0.8–0.9 T. After annealing the as-sintered magnets at ~520–600°C, coercivity increases by ~25–30% [8–20]. Typical microstructure of Nd-Fe-B sintered magnets, shown in Figure 1 is comprised of Nd_2_Fe_14_B grains separated with thin intergranular phase. In addition, secondary Nd-rich phase grains including metallic Nd, NdO_x_, and NdFe_4_B_4_ exist in the microstructure. BSE-SEM observations by Vial et al. convincingly showed the coercivity increase upon post-sinter annealing was attributed to the formation of a continuous intergranular phase to isolate Nd_2_Fe_14_B grains [12]. This intergranular phase was assumed to be Nd-rich based on the contrast in the BSE-SEM images. The structure of the intergranular phase was identified to be amorphous by Li et al. using high-resolution TEM as shown in Figure 2(a,c) for the as-sintered and optimally post-sinter annealed samples [19]. The mechanism of the formation of the intergranular phase was elaborated by Sepehri-Amin et al. to be the Nd/NdCu eutectic reaction; using three-dimensional atom probe (3DAP), they found NdCu precipitates coexist with metallic Nd at the triple junctions of the as-sintered magnet, which melt at the temperatures above 520°C during post-sinter annealing and infiltrate into the grain boundaries to form thin intergranular phase [21].

Reproduced by permission from [28], copyright (2016, Elsevier).

Although the presence of the intergranular phase was known from the early study of Nd-Fe-B sintered magnets [12,22], its chemical composition and the magnetism have not been determined until recently. The 3DAP study by Sepehri-Amin et al. showed the grain boundaries in as-sintered magnet has only slight segregation of Nd without forming any distinct phase. However, a distinct amorphous phase was observed along the grain boundaries in the post–sinter annealed samples with an average thickness of ~3 nm. This phase was identified to be amorphous containing over 65 at.% of Fe and Co as shown in Figure 2(b,d) [21]. In order to estimate the magnetization of the intergranular phase, a thin film with the identical chemical composition as the intergranular phase determined by 3DAP, Nd_29.9_Fe_65.8_B_3.1_Cu_1.2_, was prepared. The amorphous film was found to be ferromagnetic with the magnetization of 0.6 T [21]. This work suggested that the Nd_2_Fe_14_B grains in optimally annealed sintered magnets are exchange coupled through the thin ferromagnetic intergranular phase.

**Figure 3.**
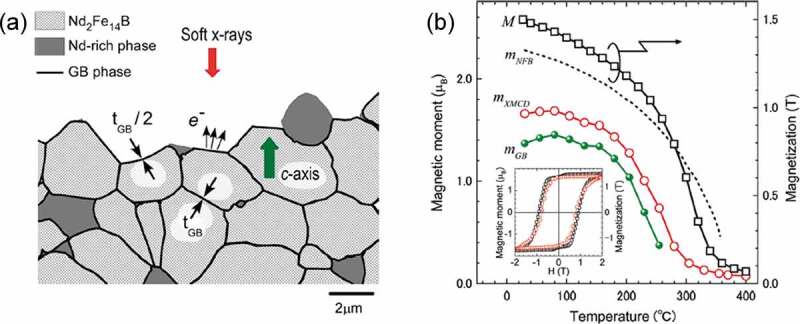
(a) Schematic diagram of XMCD measurement on fractured surface of magnet. (b) Temperature dependence of Fe magnetic moments in the GB phase (*m*_GB_) estimated for *t*_GB_~ 3 nm. *m*_XMCD_ is the Fe magnetic moment, *m*_NFB_ corresponds to the magnetic moment averaged over Fe sites in Nd_2_Fe_14_B crystal. The inset represents the magnetic field dependences of *m*_XMCD_ (open circles) and *M* (open squares) recorded at 30°C

Motivated by this work, direct measurements of the magnetism of the thin intergranular phase of Nd-Fe-B sintered magnets were carried out using various techniques. Nakamura et al. employed surface-sensitive soft X-ray magnetic circular dichroism (XMCD) to intergranularly fractured surface of Nd-Fe-B sintered magnets and found that the intergranular phase in the sintered magnets have a saturation magnetization of ~1.0 T at room temperature with a Curie temperature of ~260°C as shown in Figure 3 [23]. Kohashi et al. [24] and Murakami et al. [25] also reported ferromagnetic nature of the intergranular phase in Nd-Fe-B sintered magnets using spin-polarized scanning electron microscopy and electron holography, respectively. Note that the absolute value of the magnetization of the intergranular phase changes depending on the measurement technique such as XMCD, electron holography, or spin-polarized SEM which can originate from experimental errors in each technique and/or the fluctuation in the composition of the studied intergranular phase. However, all these measurements agree with each other on the ferromagnetic nature of the intergranular phase in Nd-Fe-B sintered magnets. Based on these studies, the unexpectedly low coercivity of Nd-Fe-B sintered magnets is concluded to be due to the ferromagnetic nature of the intergranular phase which can work as only weak pinning sites against magnetic domain wall (DW) motions during demagnetization process. Micromagnetic simulation results predict that the formation of nonferromagnetic intergranular phase which can exchange-decouple Nd_2_Fe_14_B grains would lead to a substantial increase of the coercivity of the sintered magnets [26,27].

Reproduced by permission from [23], copyright (2014, AIP Publishing).

**Figure 4.**
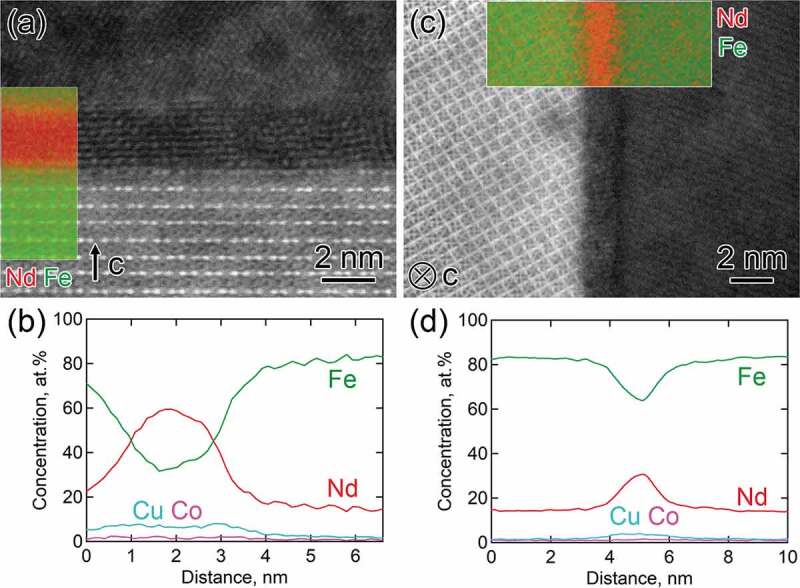
HAADF-STEM images, super imposed STEM-EDS maps, and composition line profiles obtained from EDS maps showing intergranular phase in Nd-Fe-B sintered magnets located (a, b) at the c-plane and (c, d) at the side plane of Nd_2_Fe_14_B grains. c-axis in Nd_2_Fe_14_B grain is shown with a white arrow

**Figure 5.**
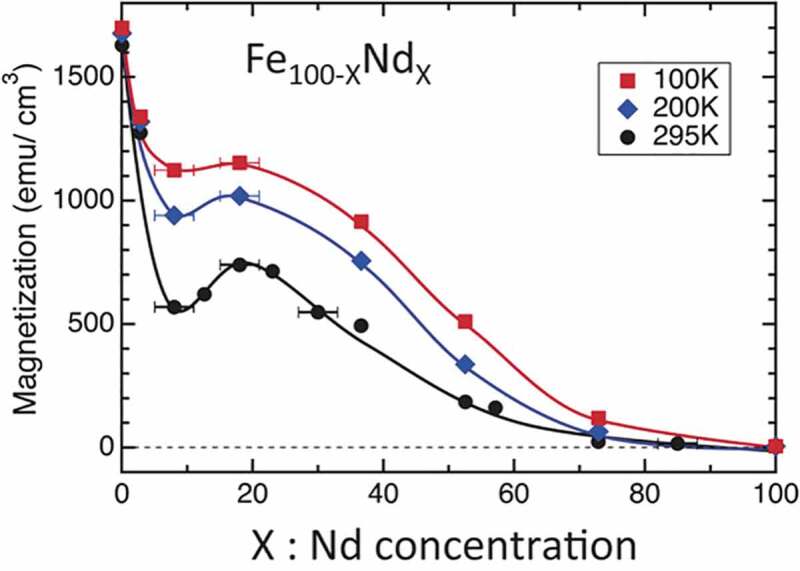
Saturation magnetization of Fe100−_*x*_Nd_*x*_ films as a function of Nd content at the temperatures of 100, 200, and 295 K

Using high angle annular dark field (HAADF) STEM and STEM-EDS studies, Sasaki et al. showed that the chemical composition of the intergranular phase in Nd-Fe-B sintered magnets varies depending on their angles with respect to the easy axis of the Nd_2_Fe_14_B grains; more enriched with Nd in the grain boundaries located perpendicular to the c-axis of Nd_2_Fe_14_B grains while its Nd content is lower in the intergranular phase formed parallel to the *c*-axis of the grains (Figure 4) [28]. Such variations of the composition of the intergranular phase are expected to influence its magnetization. Based on thin film experiments, Sakuma et al. showed that the amorphous Fe_1-*x*_Nd*_x_* phase is ferromagnetic for *x* < 0.7 in agreement with first-principles calculation results, indicating Nd concentration higher than 70% is required to decouple the intergranular exchange (Figure 5) [29].

Reproduced by permission from [28], copyright (2016, Elsevier).

Reproduced by permission from [29], copyright (2016, IOP Publishing).

**Figure 6.**
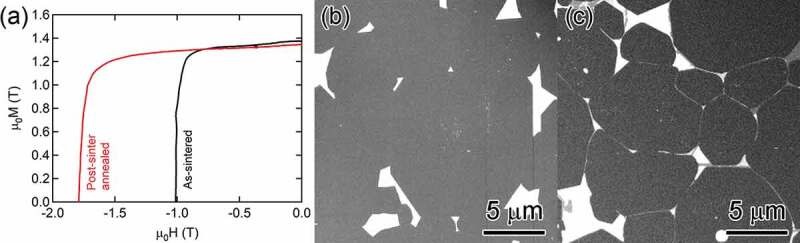
(a) Demagnetization curves for as-sintered and post-sinter annealed Ga-doped Nd-rich Ga-lean Nd-Fe-B sintered magnets. Backscattered electron SEM images taken from (b) as-sintered and (c) post-sinter annealed samples

The addition of a small amount of Ga to Nd-Fe-B sintered magnets has been known to increase the coercivity by improving the wettability of the intergranular phase during post-sinter annealing [30–33]. In earlier development of Nd-Fe-B sintered magnets, priority has been given to the achievement of high remanence. Then, part of Nd was replaced with Dy for increasing the coercivity. As a result, the chemical composition of the N50 type sintered magnet was around Nd_13.9_Fe_79.5_B_6_Cu_0.1_, which is slightly richer in Nd with respect to the stoichiometry of Nd_2_Fe_14_B, Nd_11.7_Fe_82.4_B_5.8_. In order to increase the coercivity of the Nd-Fe-B sintered magnet without using Dy, Nakajima et al. developed a Nd-rich and B-lean alloy with a trace Ga addition, i.e. Fe_77.6_RE_14.8_B_5.1_Cu_0.1_Ga_0.5_ as strip cast alloy for Nd-Fe-B sintered magnet with a substantially improved coercivity of 1.8 T for the grain size as large as ~5 µm [34]. Microstructure investigations by Sasaki et al. (Figure 6) showed the formation of three types of nonferromagnetic intergranular phases (Ia$\overset{ˉ}{3}$, amorphous Nd-rich, and Nd_6_Fe_13_Ga phases) are the main reason for the substantial enhancement of coercivity to 1.8 T upon post-sinter annealing [35]. Niitsu et al. also [36] also reported these three types of the intergranular phases are nonferromagnetic using electron holography. Magneto-optical Kerr effect microscopy by Soderznik et al. showed that the formation of thick non-ferromagnetic intergranular phase suppressed the cascade propagation of magnetic domains during the demagnetization process, which was considered to be responsible for the enhancement of coercivity [37].

Reproduced by permission from [35], copyright (2016, Elsevier).

In short, low coercivity, 0.2 *H*_A_, of the Nd-Fe-B sintered magnet with respect to the anisotropy field of the Nd_2_Fe_14_B phase is due to the intergranular exchange coupling through ferromagnetic intergranular phase.

## 3. Why does grain size reduction lead to increase in *H*_c_?

**Figure 7.**
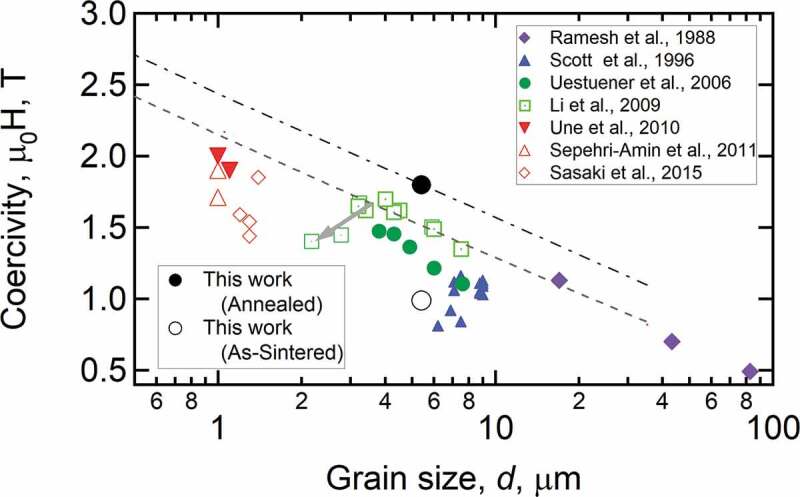
Grain size dependency of coercivity of exchange-coupled and exchange-decoupled Nd-Fe-B sintered magnets

**Figure 8.**
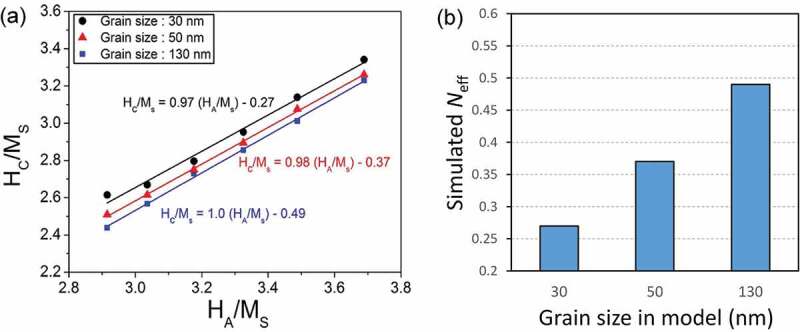
(a) *H*_c_/*M*_s_ as a function of *H*_A_/*M*_s_ of the micromagnetic models with an average grain size of 30, 50, and 130 nm. The micromagnetic parameters of *α* and *N*_eff_ are measured from the slope and intercept of a linear fit to this graph [44]. (b) Simulated *N*_eff_ at different modeled grain sizes

**Figure 9.**
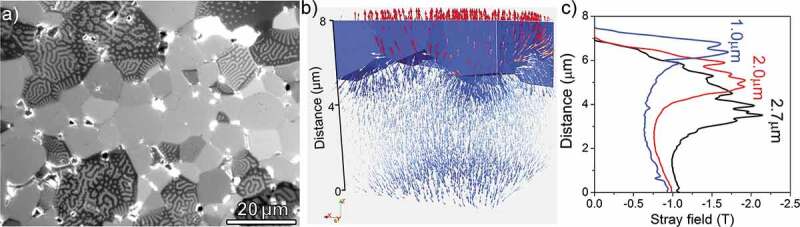
(a) Magneto optical Kerr effect (MOKE) microscopy of the surface of Nd-Fe-B sintered magnet in the remanent state after full magnetization under 5 T magnetic field and (b) simulations of stray field from the surface grain to the interior grains in anisotropic sintered magnet and (c) calculated maximum stray field from cross-sectional slices at *Z* = 0 to *Z* = 8 µm for different grain sizes

It is well known that the coercivity of anisotropic Nd-Fe-B-based magnets increases as grain size reduces as shown in Figure 7 [35,38–40]. The reason for this linear coercivity increase upon reduction of grain size, expressed as *H*_c_ = *a* – *b* ln*D* by Ramesh et al. [38], was explained due to the reduction of defect densities in exchange decoupled Nd_2_Fe_14_B grains. However, as described above, the recent investigations showed that the Nd_2_Fe_14_B grains are exchange coupled in anisotropic Nd-Fe-B sintered magnets and the reduction of defect densities cannot explain the grain size dependence of coercivity. Moreover, coercivity decrease was reported for the grain size below 3 µm deviating from the linear grain size dependence of coercivity (Figure 7). Li et al. reported the reduction of the coercivity in the fine grain-sized Nd-Fe-B sintered magnets produced by conventional powder processing routes is due to the oxidation of metallic Nd during the sintering process resulting in the lack of Nd-rich intergranular phase [41]. As discussed in the previous section, metallic Nd coexisting with NdCu is necessary for the uniform formation of the intergranular phase. When metallic Nd is oxidized, the eutectic melting of Nd/NdCu does not occur during the post-sinter annealing process. In order to suppress the reduction of coercivity in *D* < 3 µm, the oxidation of the metallic Nd phase during the powder metallurgy process must be suppressed. To overcome this problem, Une et al. prepared ultrafine grained (*D* < 1 µm) Nd_2_Fe_14_B/Nd powders by He-jet milling and sintered the powders using the press-less sintering process in Ar atmosphere [42]. This led to the realization of ultrafine grained sintered magnets with *D* ~ 1 µm, and the coercivity of 2 T was demonstrated without alloying any HRE elements. A similar trend of grain size dependence of coercivity was also reported in the Ga-doped Nd-rich B-lean Nd-Fe-B sintered magnets in which a better magnetic isolation of Nd_2_Fe_14_B grains is realized via the formation of Nd_6_Fe_13_Ga intergranular phase as shown in Figure 7 [35]. However, the underlying physics for this grain size dependence of coercivity had not been explicitly explained. Based on the 2D micromagnetic simulations, Schrefl et al. showed a long-range magnetic interaction exists among Nd_2_Fe_14_B grains which becomes less effective as grain size decreases; as a result, the demagnetization field decreases in the smaller grain-sized magnets [43]. Sepehri-Amin et al. employed 3D micromagnetic simulations and qualitatively reproduced the grain size dependence of coercivity in the exchange-coupled anisotropic Nd-Fe-B sintered magnets [44]. Coercivity is often expressed as *H_c_*(*T*)* = αH*_A_(*T*) – *N*_eff_*M*_s_(*T*) in which the first term is the influence of the defects and grain misalignment on the anisotropy field and the second term is related to the reduction of coercivity due to the demagnetization field [45]. Micromagnetic simulations on the exchange-coupled Nd-Fe-B sintered magnets have shown that improved coercivity by the reduction of the grain size is due to the lower effective demagnetization constant, *N*_eff_, as shown in Figure 8. This means that the effective demagnetization field, *N*_eff_*M*_s_, decreases as the grain size decreases, giving rise to a larger coercivity for smaller grain size. In the Nd-Fe-B sintered magnets, due to the surface machining or oxidations, the surface grains have reduced anisotropy field and hence almost no coercivity. Hence, the surface grains become multidomain at a remanent state and their magnetization reversal occurs at a much lower magnetic field than the nucleation field during the demagnetization process as often observed in the magneto optical Kerr effect microscopy (shown in Figure 9(a)). XMCD measurement by Billington et al. [46] also showed the multidomain grains with small coercivity on the polished surface. Nevertheless, the surface coercivity of the fractured surface on which Nd-rich intergranular phase still exist show comparable coercivity to that of bulk value [46]. The reversed grains on polished surface cause stray field to the surrounding grains. Sepehri-Amin et al. calculated the stray field generated by the reversed surface grain [44]. The distribution of the demagnetization vectors shown in Figure 9(b) and calculated distribution of the stray field at the surrounding of the reversed grains is shown in Figure 9(c). These simulations showed that the coercivity increase with decreasing grain size is due to the reduction in the effective stray field arising from the neighboring grains. Upon reduction of the grain size, the maximum stray field which is at the interface with the reversed neighboring grains decreases. Larger stray field produced by the larger sized grains assist the magnetization reversal of the neighboring grains which is detrimental to coercivity.

Reproduced by permission from [35], copyright (2016, Elsevier).

Reproduced by permission from [44], copyright (2014, Elsevier).

Reproduced by permission from [44], copyright (2014, Elsevier).

In short, early and more recent micromagnetic simulations works for polycrystalline anisotropic magnets confirmed the grain size dependence of the coercivity of anisotropic sintered magnets is explained by the reduction of stray field with decreasing grain size.

## 4. Why does HRE grain boundary diffusion process increase *H*_c_?

Since the first report by Park et al. [47], grain boundary diffusion (GBD) process using HRE elements has been developed as an HRE-efficient technique to enhance the coercivity of Nd-Fe-B magnet. In this process, Nd-Fe-B sintered magnets are coated with Dy or Tb in the form of oxide, fluoride, or pure metals then undergo a subsequent heat treatment [48–52], i.e. heat-treated at elevated temperature of 800–1000°C for 3–10 h followed by an optimum post-sinter annealing which results in the diffusion (infiltration) of HRE into the magnet and the formation of HRE-enrich shell in Nd_2_Fe_14_B grains [53,54]. The coercivity of the magnet is increased due to the formation of HRE-enriched high-*H*_A_ shell; meanwhile, the loss of remanent magnetization due to the antiferromagnetic coupling of Dy with Fe in the 2:14:1 phase is minimized since only a small amount of HRE is locally utilized in the shell of the 2:14:1 grains. Although the GBD process is now widely used to manufacture high-coercivity Nd-Fe-B sintered magnets for traction motor and wind turbine applications, the underlying mechanism for the formation of unique HRE-rich shell structure and the upper limit of coercivity using this method have not been well understood.

**Figure 10.**
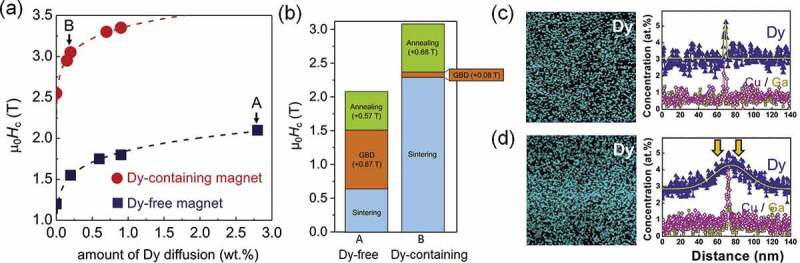
(a) Variations in coercivity as functions of the amount of Dy diffusion for GBD processed Dy-free and Dy-containing magnets. (b) Δµ_0_*H*_c_ of samples A and B in (a) with GBD process steps (GBD treatment and post-diffusion annealing). EDS elemental map of Nd and EDS line profiles across GB phase in (c) GBD treated and (d) post-diffusion annealed Dy-free magnet

In order to understand the effect of coercivity increase in the course of the entire diffusion process including the Dy-diffusion and subsequent heat-treatment process, Kim et al. investigated the coercivity change in each step for Dy-free and Dy-containing samples and their microstructure change in details [55]. Figure 10(a) shows the coercivity as function of the amount of Dy diffusion applied to two types of initial samples: one is Dy-free Nd-Fe-B and the other is Dy-containing Nd_24.3_Dy_7.5_Fe_66.0_B_1.0_Cu_0.1_Al_0.15_Co_0.9_Ga_0.05_ (wt.%) sintered magnets. Coercivity increases as the amount of Dy supply increases and it eventually reaches saturation. As shown in Figure 10(b), when GBD process is applied to the Dy-free sample, the coercivity does not increase to more than 2.1 T. On the other hand, when 0.1 wt.% Dy is diffused to the Dy-containing sample with the initial coercivity of 2.3 T, the coercivity reaches 3 T. In order to reduce the amount of Dy from the GBD processed high coercivity magnet, it is desirable to increase the coercivity to higher than 3 T without alloying Dy in the starting sample. It is also known that the high coercivity can be achieved only when the sample is optimally heat-treated after the diffusion process [55]. Based on the STEM-EDS elemental analyses as shown in Figure 10(c,d), they found that post-annealing after Dy-diffusion leads to the formation of a secondary Dy-rich shell, which indicates a solid diffusion of Dy atoms from the GB to the shell region during the annealing process. As a result, the Nd concentration in the intergranular phase increased substantially compared to the as GBD treated sample because the Nd atoms rejected from the Dy-rich shell are enriched in the intergranular phase, which also leads to the formation of thicker intergranular phase with higher Nd concentration.

Reproduced by permission from [55], copyright (2019, Elsevier).

**Figure 11.**
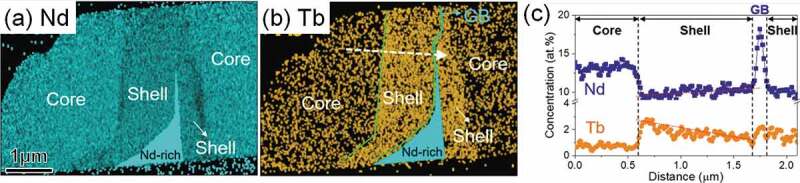
EDS elemental map for (a) Nd and (b) Tb. The line scan profile of Tb across the shell (dashed line in Tb map) is shown in (c)

**Figure 12.**
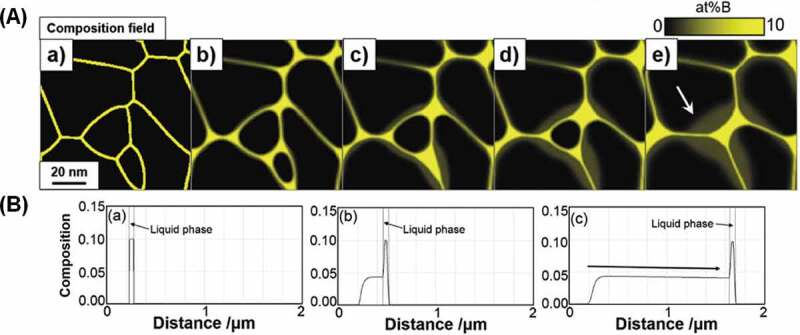
(a) 2D phase-field simulation of the microstructure changes based on the CILFM mechanism during isothermal ageing for (a) *t*’ = 0, (b) *t*’ = 0.5, (c) *t*’ = 2.5, (d) *t*’ = 5, and (e) *t*’ = 10 (*t*’ is dimensionless time). (b) 1D phase-field simulation of the real scale calculation on the shell formation process during isothermal ageing for (a) *t*’ = 0, (b) *t’* = 5 × 10^3^ , and (c) *t*’ = 3 × 10^4^ (*t*’ is dimensionless time)

One of the long-standing questions on the HRE GBD process was the formation mechanism of a unique shell structure that is asymmetric with respect to grain boundaries. Seelam et al. [56] proposed that the HRE-rich shell is formed by the solidification of liquid phase during cooling from the processing temperature, thereby explaining the sharp core/shell interface and faceted core shape. However, this does not explain the anisotropic nature of the HRE-rich shell around grain boundaries and apparently large fraction of the HRE-rich shell near the surface of GBD processed magnets. Recently, Kim et al. investigated the formation mechanism of Tb-rich shell in a Tb-diffusion processed Nd-Fe-B sintered magnet based on detailed microstructure characterization using STEM and phase-field simulation [57]. As shown in Figure 11, the formation of Tb-rich shell for the former is always asymmetrical along the GB. Inside the shell, the concentration of Tb gradually increases from the GB side to the core/shell interface then abruptly drops in the core range (see Figure 11(c)). Further TEM observation showed the existence of the stacking faults at the core/shell interface [57]. To explain these microstructure features, Kim et al. proposed the chemically induced liquid film migration (CILFM) mechanism to explain the Tb-rich shell formation. As shown in the phase-field simulation (Figure 12(a)), the grain grows by the migration of liquid film GB at the processing temperature. A continuous decrease of Tb concentration from core/shell interface to grain surface is then found in the shell as shown in Figure 12(b) in agreement with the experimental observation.

Reproduced by permission from [55], copyright (2019, Elsevier).

Reproduced by permission from [57], copyright (2020, Elsevier).

In short, the coercivity enhancement of HRE GBD processed magnets are not only due to the formation of the HRE-rich shell, but also due to the formation of the Nd-rich intergranular phase by the optimal post-diffusion annealing. The HRE-rich shell is formed by the CILFM, which explains the asymmetric feature of the HRE-rich shell.

## 5. How to increase the coercivity of hot-deformed magnets using HRE diffusion bypassing grain coarsening?

**Figure 13.**
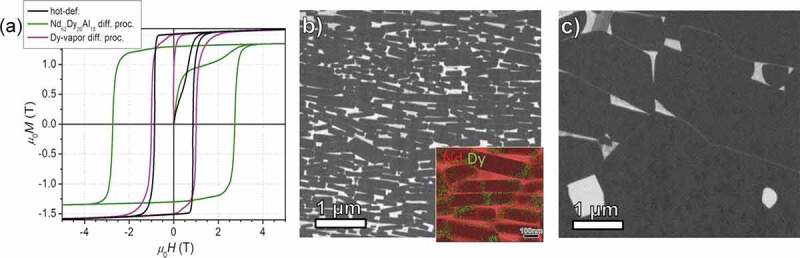
(a) Magnetization curves of hot-deformed, Nd-Dy-Al diffusion-processed and Dy-vapor diffusion-processed magnet samples. (b) BSE-SEM image of Nd_62_Dy_20_Al_18_ eutectic diffusion-processed hot-deformed magnet. The inset shows the TEM-EDS elemental mapping image. (c) BSE-SEM image of Dy-vapor diffusion-processed hot-deformed magnet

HRE GBD is expected to increase the *H*_c_ of hot-deformed magnet as well. However, the conventional HRE GBD process applied on sintered magnets fail on the hot-deformed magnets as shown in Figure 13(a), where Dy-vapor diffusion gives rise to negligible increases of *H*_c_ as the result of the coarsening of grains at the high processing temperature of ~900°C. In order to reduce the process temperature to suppress the grain coarsening (see Figure 13(c)) of the hot-deformed magnets, Sepehri-Amin et al. [58] applied a low-temperature eutectic diffusion process. This method is an extension of the Nd-Cu eutectic diffusion process that was invented to enhance the coercivity of HDDR powder [59] as well as hot-deformed magnets [60] without using HRE. High *H*_c_ value of 2.3 T or 2.5 T was reported after the diffusion with Nd_70_Cu_30_ [60] or Nd_90_Al_10_ [61] alloys. By adding HRE to the eutectic alloys like Nd_60_Dy_20_Cu_20_ [58], Nd_62_Dy_20_Al_18_ [62] and Nd_60_Tb_20_Cu_20_ [63], the coercivity was further enhance to 2.6–2.8 T at a processing temperature ≤700°C accompanied by a loss of remanence. This loss of remanence is more serious compared to the conventional HRE GBD process applied to sintered magnets, which is attributed to the formation of thick Nd-rich nonferromagnetic intergranular phases observed in the eutectic diffusion processed hot-deformed magnets. Although the formation of HRE-rich shell was found, only a few grains are fully enveloped by HRE-enriched shell in Figure 13(b), and most of the platelet-shaped grains are partly covered either on the sides or on the *c*-plane surfaces [62,63]. Micromagnetic simulations [63] suggested that these high-*H*_A_ shell can obstruct the propagation of magnetic DWs if the 2:14:1 grains are exchange coupled, or can force a relocation of nucleation site from grain surface to the core/shell interface if the grains are exchange decoupled.

Reproduced by permission from [62], copyright (2017, Elsevier).

In short, GB infiltration of low melting temperature Nd-HRE-(Cu,Al) eutectic alloys leads to a substantial increase of coercivity of hot-deformed magnets without inducing grain coarsening.

## 6. What is the coercivity mechanism of Nd-Fe-B magnets?

**Figure 14.**
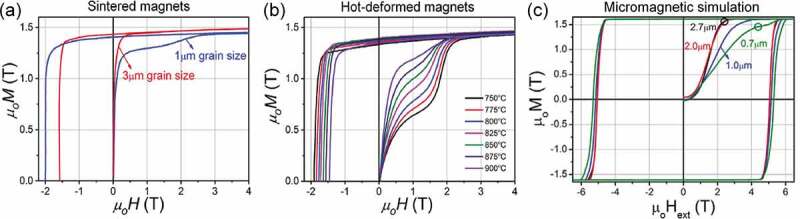
(a) Initial and demagnetization curves of sintered magnet with different grain sizes [66]. (b) Initial and demagnetization curves of hot-deformed magnet with different grain sizes [67]. (c) Simulated initial curves and hysteresis loops of models with different grain sizes [44]

Reproduced from Sepehri-Amin et al. [44,66] and Liu et al. [67] with copyright permissions from Elsevier.

The magnetization reversals in permanent magnets should at least consist of two steps: the nucleation of reversed domain and the propagation of DW throughout the matrix. Depending on which step controls the magnetization reversal, the coercivity mechanism is generally described as nucleation-control or DW pinning-control. In early studies of Nd-Fe-B sintered magnets with a large grain size of ~5–10 µm, the measured initial magnetization curve from a thermally demagnetized state always shows a large susceptibility, and the magnetization is easily saturated after applying a small external magnetic field. This ‘one-step’ initial magnetization curve was explained as evidence for the lack of effective DW pinning sites, indicating the nucleation-control reversal mechanism [45,64]. However, Hadjipanayis et al. argued that the ‘one-step’ feature only demonstrated the lack of pinning site inside the large-size grains, while pinning of DW along grain boundaries is still possible [65]. Evidence for the latter has been found in the recently developed sintered magnet with an ultra-fine grain size of 1 µm [66] and in the hot-deformed magnet with submicron grain size [67]. These grain sizes are comparable to the domain size of thermally demagnetized Nd_2_Fe_14_B so after initial DW displacements in the matrix, they are pinned at grain boundaries. This gives rise to the ‘two-step’ initial magnetization curves consisting of initial high susceptible region in the low magnetic field, where DWs in multidomain size grains displaces, and the susceptibility become low as the DW are pinned at grain boundaries. When the DWs are de-pinned, magnetization again increases to saturation as shown in Figure 14(a,b). The micromagnetic simulations on the grain size dependence of initial curve by Sepehri-Amin et al. reproduced the two-step initial magnetization curves as shown in Figure 14(c) [44], where ‘one-step’ initial curve transfers to ‘two-step’ shape when grain size reduces smaller than 1 µm. Note that the grain size where the ‘two-step’ feature appears for both experimental curves and simulation curves is apparently larger than the conventionally defined single-domain size, $D_{c}=\frac{72\sqrt{AK}}{\mu_{0}M_{s}^{2}}\approx 200nm$ for Nd_2_Fe_14_B, which is valid to determine the single-domain size of a magnetized particle [68] but improper for the discussion of ‘two-step’ feature here.

**Figure 15.**
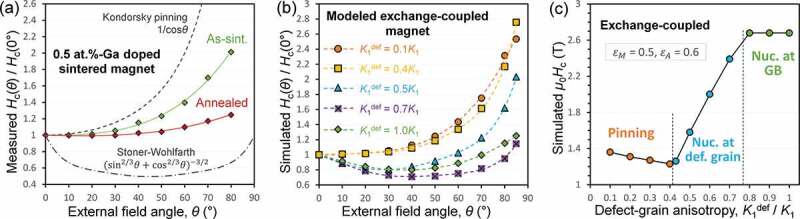
(a) Angular dependence of coercivity of Ga-doped sintered Nd-Fe-B magnet with or without post-sinter annealing. (b) Simulated angular dependence of coercivity of a modeled polycrystalline Nd-Fe-B magnet. (c) The simulated $H_{c}\left(0^{\circ }\right)$ corresponding to (b), where the reversal mechanism is controlled by the anisotropy energy of the defect grain, $K_{1}^{def}$

Another method to characterize the magnetization reversal mechanism in the ferromagnetic materials is based on angular dependence of coercivity [69–73]. Stoner–Wohlfarth model [74], which describes the coherent reversal with uniaxial anisotropy energy $K_{u}=K_{1}sin^{2}\theta$, gives a $H_{c}\left(\theta \right)$ curve with a local minimum at $\theta =45^{\circ }$. Another model named after Kondorsky to describe the DW depinning process gives $H_{c}\left(\theta \right)=\frac{E_{b}}{cos\theta }\propto \frac{1}{cos\theta }$ that assumes a fixed energy barrier$E_{b}$ despite the change of field angle θ. Micromagnetic simulations [75,76] show that the simulated $H_{c}\left(\theta \right)$ of a large size system does not strictly follow the results of simple model. However, the shape of $H_{c}\left(\theta \right)$ curve still correlates strongly with coercivity mechanism. Bance et al. [75] showed the shift of $H_{c}\left(\theta \right)$ curve from Stoner–Wohlfarth-like to Kondorsky-like by reducing the thickness of soft defect layer in a single Nd_2_Fe_14_B grain model. However, this simulation was carried out on a single crystal model. For simulating the magnetization behaviors of actual permanent magnets, the model must be exchange-coupled anisotropic polycrystals. Li et al. [76] showed a direct correlation between reversal mechanism and the appearance of local minimum in $H_{c}\left(\theta \right)$ based on the simulations of a polycrystalline model (see Figure 15(b,c)). Experimentally measured $H_{c}\left(\theta \right)$ of both conventional sintered and hot-deformed magnets are found to monotonically increase with θ, reaching a factor of $H_{c}\left(80^{\circ }\right)/H_{c}\left(0^{\circ }\right)$ as 1.6–2.0 [70–73,75], indicating that DW pinning process is dominant for the magnetization reversal of the exchange-coupled magnets. An analogous phenomenon which may correlate with the angular dependence of coercivity is that the coercivity of Nd-Fe-B magnet monotonically decreases with the degree of grain alignment [77], which is also explained in simulation by Fujisaki et al. [78] as the result of DW pinning at grain boundaries. Interestingly, for those GB-engineered magnets with a large fraction of Nd-rich nonferromagnetic intergranular phases, the pinning feature of $H_{c}\left(\theta \right)$ becomes weak while nucleation feature tends to emerge. For example, a very slowly increasing $H_{c}\left(\theta \right)$ was observed for the optimally annealed Ga-doped Nd-rich B-lean sintered magnet (Figure 15(a)), and a slowly varying $H_{c}\left(\theta \right)$ with a weak ‘dip’ was observed for the hot-deformed magnet with a heavy infiltration of Nd-Cu alloy [75]. These slowly varying $H_{c}\left(\theta \right)$ deviates from a monotonically and sharply increasing $H_{c}\left(\theta \right)$ observed in the simulated pinning-control reversal, and also largely deviate from that of an ideally exchange-decoupled model which shows Stoner–Wohlfarth-like curve [76], indicating that the mechanism of $H_{c}$ here should not be simply described as pinning- or nucleation-control. Therefore, from the angular dependence of *H*_c_, it is concluded that the *H*_c_ mechanism is typical pinning type for the exchange-coupled sintered magnet and hot-deformed magnet. Micromagnetic simulation indicates that the mechanism would change to the nucleation type when grains are perfectly exchange decouples, while such exchange-decoupled magnets have not been demonstrated in the Nd-Fe-B system.

Reproduced by permission from [76], copyright (2020, Elsevier).

In short, the coercivity of the exchange-coupled Nd-Fe-B magnets can be explained by the DW pinning at the grain boundaries. The absence of the pinning feature in the initial magnetization curve is merely due to the multidomain state of large grains of the standard sintered magnets. When grain size becomes comparable to the domain size (~1 µm), the DW pinning by grain boundaries become visible in the initial magnetization curve. When grains are exchange decoupled by the formation of nonferromagnetic intergranular phase, the magnetization mechanism is expected to transfer to the nucleation mechanism, by which higher coercivity will be achieved.

## 7. How to improve the thermal stability of *H*_c_?

**Figure 16.**
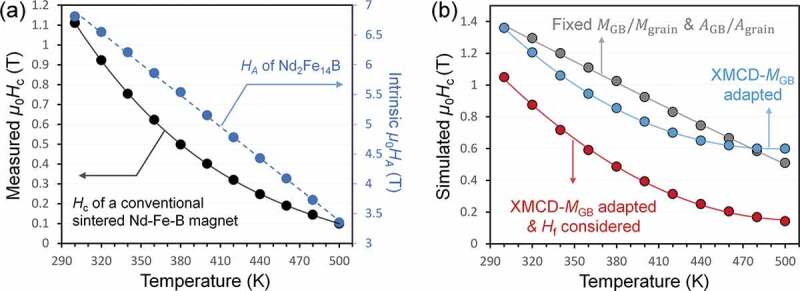
(a) Comparison of *H*_A_(*T*) and *H*_c_(*T*) of a conventional Nd-Fe-B sintered magnet. (b) Simulated *H*_c_(*T*) of a modeled sintered magnet

High-temperature application is always a challenge for Nd-Fe-B magnet due to the severe reduction of *H*_c_ at elevated temperature. One long-standing question about the temperature dependence of coercivity is its nonlinear change with concavity downwards with respect to the change of *H*_A_(*T*) as shown in Figure 16(a). The discrepancy between *H*_c_(*T*) and *H*_A_(*T*) cannot be explained with the well-known equation of coercivity, $H_{c}=\alpha H_{A}-N_{eff}M_{s}$, which predicts linear correlation between *H*_c_(*T*) and *H*_A_(*T*) since microstructure parameter α was assumed to be constant. Recently, Li et al. [79] reproduced the concavity of *H*_c_(*T*) in the micromagnetic simulations (Figure 16(b)) that considered the temperature dependence of GB magnetization adapted from XMCD measurements [23]. It is explained that the parameter α is also temperature-dependent since the GB magnetization follows different temperature dependence with respect to that of Nd_2_Fe_14_B. Following this argument, the magnet with reduced GB magnetization should exhibit linear change of *H*_c_(*T*) [79]. In fact, the linear *H*_c_(*T*) has been reported in several works on eutectic diffusion processed hot-deformed magnet [61–63] without any insight on the underlying mechanism.

Reproduced by permission from [79], copyright (2020, Elsevier).

**Figure 17.**
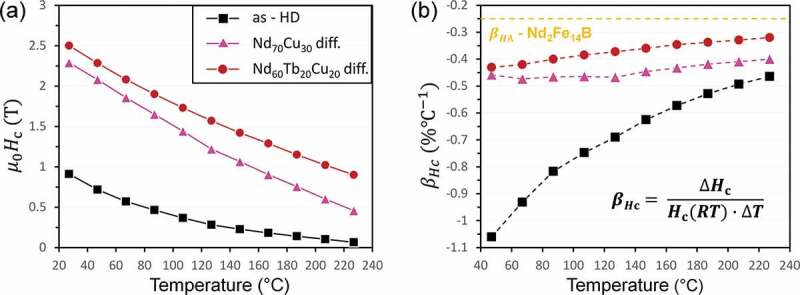
(a) Temperature dependence of coercivity of as-hot-deformed and eutectic diffusion-processed Nd-Fe-B magnets [63]. (b) The temperature coefficient of coercivity for samples in (a)

Conventionally, the thermal stability of coercivity of Nd-Fe-B magnet is quantitatively described by its temperature coefficient, $\beta_{Hc}=\frac{\Delta H_{c}}{H_{c}\left(RT\right)\Delta T}$, where a small negative value of $\beta_{Hc}$ guarantees a small relative change (reduction) of *H*_c_. The typical value of $\beta_{Hc}$ for Dy-free sintered magnet is ~ −0.6% K^−1^ at 160°C. An important feature about $\beta_{Hc}$ is that, like coercivity, it is microstructure controlled and thus can be improved via magnet processing. By substituting Kronmüller’s equation in the expression of $\beta_{Hc}$ [63,80], it is found that improved value of $\beta_{Hc}$ can be achieved in a microstructure with large value of α or small value of $N_{eff}$. Grain size reduction is one way to improve the thermal stability of coercivity as demonstrated by Liu et al. [67] which originates from the reduction of $N_{eff}$ in smaller grain-sized magnets. In hot-deformed magnets, *N*_eff_ can be reduced by decreasing the aspect ratio of the platelet-like grains [81], which lead to a moderate improvement of $\beta_{Hc}$ [82]. On the other hand, large α value means the diminish of the defects with low anisotropy field such as ferromagnetic grain boundaries or soft-magnetic intergranular phase. Improvement of $\beta_{Hc}$ via increased α has been demonstrated in GB engineered Nd-Fe-B magnets, e.g. the Ga-doped Nd-rich B-lean sintered magnet after optimized annealing process [76], or the hot-deformed magnets that were diffusion processed with Nd*_x_*M*_y_* (M = Cu, Al, Ga et al.) eutectic alloys [60,61,83–85]. The Nd-Cu eutectic diffusion process applied to a hot-deformed magnet improved $\beta_{Hc}$ to around −0.40% K^−1^ at 500 K (see Figure 17(a,b)). Tang et al. [86] demonstrated that the application of the Nd-Cu diffusion process to (Nd_0.8_Ce_0.2_)-Fe-B hot-deformed magnet also led to a substantial improvement of the thermal stability of *H*_c_, giving rise to the higher *H*_c_ at elevated temperature that is superior to that of Nd-Fe-B magnets.

Reproduced by permission from [63], copyright (2018, Elsevier).

The formation of core–shell structure in HRE eutectic diffusion processed magnet was also found to be effective in improving the thermal stability of coercivity. Benefiting from the good thermal stability of *H*_A_ of Tb_2_Fe_14_B phase, Nd_60_Tb_20_Cu_20_ eutectic diffusion processed Nd-Fe-B hot-deformed magnet was found to show substantially improved $\beta_{Hc}$ of −0.3% K^−1^ (500 K) as shown in Figure 17(b) [63], much superior to that of Nd_70_Cu_30_ eutectic diffusion processed sample. Micromagnetic simulations by Li et al. [63] suggested that the formation of Tb-rich shell improves the thermal stability of coercivity in both exchange-coupled and exchange-decoupled grain models. On the contrary, the thermal stability of Pr_80_Cu_20_ diffusion processed magnet was found inferior to that of Nd_80_Cu_20_ diffusion processed sample [87], as a result of the poor thermal stability of Pr_2_Fe_14_B *H*_A_.

In short, the thermal stability of coercivity can be improved in the same approach of improving coercivity without using HRE, i.e. reducing the grain size, tuning the grain shape and modifying the GB magnetism, and it can be further improved by introducing (Nd,HRE)_2_Fe_14_B shell in Nd_2_Fe_14_B grains. In particular, modifying GB to nonferromagnetic can suppress the concavity of *H*_c_(*T*) curve and change the shape closer to *H*_A_(T).

## 8. Why does Ce-substituted (Nd_1-*x*_Ce_*x*_)-Fe-B magnets show improved thermal stability of coercivity?

The ever-increasing demands on the Nd-Fe-B-based permanent magnets has raised concerns on the future supply chain of Nd which is the main rare-earth element used in these magnets. Although Nd is relatively abundant middle rare-earth elements, light rare earth (LRE) elements are always produced as byproducts and its effective usage is expected to reduce the entire cost of industrially useful rare-earth elements. Pathak et al. proposed the possibility of cost-effective permanent magnets by substituting part of Nd with LRE element, Ce, for Nd [88]. Since then, many investigations have been carried out to optimize the permanent magnetic properties of both isotropic and anisotropic (Nd_1-*x*_LRE_*x*_)-Fe-B magnets [89–94]. However, the intrinsic magnetic properties of LRE_2_Fe_14_B compounds are inferior to those of Nd_2_Fe_14_B, limiting the performance of LRE-substituted permanent magnets; e.g. Ce_2_Fe_14_B compound has *µ*_0_*H*_A_ = 2.6 T, *µ*_0_*M*_s_ = 1.17 T and *T*_c_ = 150°C with respect to *µ*_0_*H*_A_ = 7.5 T, *µ*_0_*M*_s_ = 1.6 T and *T*_c_ = 320°C for Nd_2_Fe_14_B [95]. Recent studies on the intrinsic magnetic properties of single crystalline (Nd1-_*x*_Ce_*x*_)_2_Fe_14_B by Susner et al. [96] have shown that the magnetization (*M*_s_/f.u.) and anisotropy field can be well preserved up to *x* = 0.22. Hence, limited substitution of LRE elements such as Ce for Nd is expected to minimize the degradation of hard magnetic properties of (Nd1-_*x*_Ce_*x*_)-Fe-B-based magnets. An early work of Zhu et al. [97] reported a coercivity of 1.2 T and remanence of 1.37 T in (Nd,Ce)-Fe-B sintered magnet prepared by so-called ‘dual alloy’ method which utilized mixed Nd-Fe-B and (Nd,Ce)-Fe-B powders for sintering. Fan et al. [98] also showed by limited usage of Ce, i.e. 25% substitution of Nd for Ce, a moderate coercivity of 1.21 T and a remanence of 1.33 T can be achieved in (Nd,Ce)-Fe-B sintered magnets. However, these moderate magnetic properties are still far to consider (Nd,Ce)-Fe-B-based permanent magnets as high-performance magnets. In addition to the intrinsic origins for the deterioration of coercivity and remanent magnetization, the formation of undesired phases such as CeFe_2_ and the lack of RE-rich intergranular phase were found to be a microstructure origin for the inferior extrinsic magnetic properties in the (Nd,Ce)-Fe-B magnets [97,99,100].

**Figure 18.**
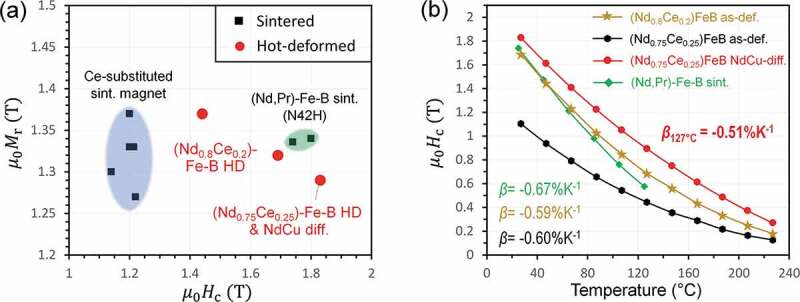
(a) Remanence versus coercivity of Ce-substituted sintered magnets with 18–27 at.% Ce substitution for Nd [97,98,102,103], and of hot-deformed magnets with 20 at.% Ce substitution for Nd. The N42H-grade sintered magnets [104] is plotted for comparison. (b) Temperature dependence of coercivity of (Nd,Pr)-Fe-B sintered magnet with RE concentration of 15 at.%, hot-deformed (Nd_0.8_Ce_0.2_)-Fe-B magnet with RE concentration of 14.5 at.%, hot-deformed (Nd_0.75_Ce_0.25_)-Fe-B magnet, and Nd-Cu diffusion processed (Nd_0.75_Ce_0.25_)-Fe-B hot-deformed magnet

Recently, TOYOTA motor corporation announced that a LRE-substituted hot-deformed magnet showed superior temperature coefficient of coercivity compared to the conventional sintered magnets [101]. However, its scientific origin has not been reported. To trace the announced result scientifically, Tang et al. [86] demonstrated a (Nd_0.8_Ce_0.2_)-Fe-B-based hot-deformed magnet exhibits the permanent magnet properties comparable to those for N42H class commercial sintered magnet as shown in Figure 18. The coercivity was further enhanced to 1.83 T by optimizing the microstructure using the Nd-Cu eutectic alloy diffusion process to the (Nd_0.75_Ce_0.25_)-Fe-B hot-deformed magnets as the result of the formation of (Nd,Ce,Cu)-rich intergranular phase. The formation of Nd-rich 2:14:1 shell covering (Nd_0.75_Ce_0.25_)_2_Fe_14_B was another microstructure feature responsible for enhancement of coercivity. Although the Curie temperature of Ce_2_Fe_14_B is much lower than that of Nd_2_Fe_14_B, the 20%Ce-substituted anisotropic magnet showed the hard magnetic properties comparable to those of N42H-grade commercial sintered magnet with a similar RE concentration of 15 at.% as shown in Figure 18(a). The Ce-substituted magnet showed a temperature coefficient of coercivity of −0.59%/^o^C, which is superior to that of N42H. The origin of the improved temperature coefficient of coercivity of the Ce-substituted hot-deformed magnets was attributed to the low magnetization of the (Nd,Ce)-enriched intergranular phase and the formation of high-*H*_A_ Nd-rich shell. These reports clearly show (Nd,Ce)-Fe-B-based hot-deformed magnets with optimized microstructure can be considered as low-cost high-performance permanent magnets [86].

 Reproduced by permission from [86], copyright (2020, Elsevier).

In short, serious degradation of coercivity and remanence by substituting Nd with Ce in Nd-Fe-B magnet can be avoided by the microstructure optimization as long as the Ce concentration ratio, Ce/RE, is controlled smaller than 30%. The origin of the improved temperature coefficient of coercivity is the formation of (Nd,Ce)-rich intergranular phase and Nd-rich shell.

## 9. What is the Nd-Fe-B magnet with ultimate performance?

The ultimate performance of coercivity and its thermal stability is expected in a magnet with maximized anisotropy factor α as well as minimized demagnetization factor *N*_eff_ to approach the limit of intrinsic *H*_A_. Factor α can be maximized by the formation of nonferromagnetic intergranular phase isolating every 2:14:1 grain, resulting in a pure nucleation-control magnetization reversal in the magnet. Reducing grain size is the conventional and practical way to minimize *N*_eff_. However, this approach failed in the magnet with submicron grain size which was produced by hydrogenation–disproportionation–desorption–recombination (HDDR) and press-less sintering process (PLP) since the thin intergranular phase is ferromagnetic [105]. This result suggests the importance of the formation of nonferromagnetic GB phase fully isolating Nd_2_Fe_14_B grains. The hot-deformed magnets which consist of submicron-sized grains show µ_0_*H*_c_ values in the range of 1.0–2.0 T that are comparable with that of sintered magnets with a grain size generally much larger than 1 µm. Although the engineering of the intergranular phase via eutectic diffusion process in the hot-deformed magnets results in a substantial enhancement of coercivity, further enhancement of coercivity is expected if the side GB phase can also become paramagnetic. Note that the current experimental knowledge has no answer on how to fully exchange decoupled Nd_2_Fe_14_B grains in the anisotropic ultra-fine grain-sized Nd-Fe-B magnets.

On the other hand, the volume fraction of intergranular phase increases with reducing grain size, which will lead to a drop of remanence if all the grain boundaries are nonferromagnetic. As a simple demonstration, the volume fraction of intergranular phase in a stack of cubit blocks is given by $f_{GB}=\frac{{\left(L/D\right)}^{3}\cdot 3D^{2}\cdot t}{L^{3}}=\frac{3t}{D}$, where *L* is the total size, *D* is the block size and *t* is the thickness of the intergranular phase. For a GB thickness of *t* = 3 nm and a requirement of limited volume fraction of GB, e.g. $f_{GB}\leq 10\%\left(\frac{M_{magnet}}{M_{2:14:1}}\geq 90\%\right),$ one has D≥90nm. This simple calculation indicates that the optimized grain size of a Nd-Fe-B magnet is in the order of 100 nm, and further reduction of grain size is not suggested since the volume fraction of the intergranular phase becomes too large and the degradation of remanence is unavoidable.

Based on the above discussion, the ultimate performance of extrinsic property is expected in a magnet with grain size around several hundred nanometers, equiaxed shape, uniformly separated by nonferromagnetic intergranular phase (i.e. exchange isolated). As a reference, micromagnetic simulation consisting of exchange-decoupled equiaxed grains with a size of 100 nm predicted a coercivity of 3.5 T (~*H*_A_/2) [79]. The formation of high-*H*_A_ shell enveloping the exchange-decoupled Nd_2_Fe_14_B grains can further improve *H*_c_ and $\beta_{Hc}$ to overcome the limit of intrinsic *H*_A_ of the Nd_2_Fe_14_B phase. Since its mechanism is to force a relocation of nucleation site from grain surface to the core/shell interface where the demagnetizing field is smaller [63], *H*_c_ and $\beta_{Hc}$ will increase gradually with the increase of shell thickness when grain size is fixed. The enrichment of Dy or Tb at the thin shell region is needed which shows intrinsic $\beta_{HA}$ comparable or even better than Nd_2_Fe_14_B.

The recent advances on the multi-scale microstructure characterizations have broadened our understanding on the coercivity mechanism of Nd-Fe-B-based magnets leading to a substantial improvement on coercivity and its thermal stability in the last decade. However, still there is a gap between the experimentally achieved coercivity (≲*H*_A_/3) and the simulation predicted coercivity in an optimal microstructure (~*H*_A_/2). It is expected to fill the gap by further reduction of the grain size and magnetic isolation of Nd_2_Fe_14_B grains with thin nonferromagnetic intergranular phase, which has not been realized yet. Materials informatics and active learning [106–109], which has not been used seriously in the permanent magnet community, can pave a way for raising novel ideas on how to further optimize the microstructure of ultra-fine grain sized anisotropic Nd-Fe-B magnets. Hence, revisiting the accumulated knowledge on the phase diagram, processing, and microstructure of Nd-Fe-B-based magnets and employing materials informatics can be tools to realize Nd-Fe-B-based permanent magnets with ultimate performance without reliance on heavy rare-earth elements.
